# HIF-1α hampers dendritic cell function and Th1 generation during chronic visceral leishmaniasis

**DOI:** 10.1038/s41598-018-21891-z

**Published:** 2018-02-22

**Authors:** Akil Hammami, Belma Melda Abidin, Krista M. Heinonen, Simona Stäger

**Affiliations:** INRS-Institut Armand-Frappier and Center for Host-Parasite interactions, 531 Boulevard des Prairies, Laval (QC), H7V 1B7 Canada

## Abstract

Inflammation, although responsible for controlling infection, is often associated with the pathogenesis of chronic diseases. *Leishmania donovani*, the causative agent of visceral leishmaniasis, induces a strong inflammatory response that leads to splenomegaly and ultimately immune suppression. Inflamed tissues are typically characterized by low levels of oxygen, a microenvironment that triggers the hypoxia-inducible transcription factor 1α (HIF-1α). Although HIF-1α plays an integral role in dendritic cell function, its involvement in the generation of protective Th1 responses against *Leishmania* has not yet been studied. Here we demonstrate that HIF-1α inhibits IL-12 production in dendritic cells, limiting therefore Th1 cell development. Indeed, depletion of HIF-1α in CD11c^+^ cells resulted in higher and sustained expression of IL-12 and complete abrogation of IL-10. Moreover, CD11c-specific HIF-1α-deficient mice showed higher frequencies of IFN-γ-producing CD4 T cells in the spleen and bone marrow and, consequently, a significantly reduced parasite burden in both organs. Taken together, our results suggest that HIF-1α expression in dendritic cells largely contributes to the establishment of persistent *Leishmania* infection and may therefore represent a possible therapeutic target.

## Introduction

A balance between inflammatory and anti-inflammatory responses is essential for the proper functioning of the immune system. An imbalance towards strong inflammation can lead to several autoimmune diseases, like arthritis; in contrast, when anti-inflammatory responses dominate, the result is immunosuppression. Pathogens are a remarkable challenge for the immune system. Indeed, they have developed several strategies to evade specific immune responses and establish a microenvironment prosperous for their growth. For instance, LCMV triggers a potent inflammatory response that leads to generalized immune suppression, while the protozoan parasite *Toxoplasma gondii* drives an anti-inflammatory response mainly by inducing IL-10 and TGFβ production^1^.

Sustained inflammation is fundamental for efficient T cell priming and pathogen clearance. The pro-inflammatory cytokine IL-12, for example, is crucial for CD8 T cell and Th1 cell priming and effector function acquisition^2^. The transcription factor IRF-5, in particular, seems to be crucial for Th1 generation^3,4^. The inflammatory milieu was also shown to control antigen sensitivity by enhancing T cell receptor signaling. Likewise, type I IFN and IFNγ also appear to be required for efficient CD8 T cells priming^5^. Despite the clear role of inflammation in positively shaping T cell responses, some exceptions were reported. Indeed, a chronic inflammatory environment negatively impacts the development of memory CD8 T cell responses^6^. Inflammation also seems to play a negative role in CD8 T cell priming in an experimental model of visceral leishmaniaisis (VL).

Visceral leishmaniasis (VL) is the most severe form of leshmaniasis. The protozoan parasite *Leishmania donovani* is one of the causative agents of the disease. In the murine model, as well as in human patients, the parasite establishes persistent infection in the spleen and bone marrow.

*L. donovani* is known to induce a strong inflammatory response, characterized by the production of high amounts of IL-6 and TNF^7^. This results in splenomegaly, TNF-mediated the splenic tissue disruption, and ultimately in immunosuppression, mainly mediated by IL-10^7^.

Chronically infected and inflamed tissues are typically hypoxic. Low oxygen tensions and tissue disruption create an environment that triggers the stabilization of the hypoxia inducible factor-1α (HIF-1α). HIF-1α stabilization can also be directly induced by various pathogens, including *Leishmania* parasites^8^. The upregulation and stabilization of HIF-1α has been reported to alter dendritic cell (DCs) functions and migratory capacity (^9^; reviewed in^10,11^). HIF-1α appears to down modulate costimulatory molecule expression and impair upregulation of the chemokine receptor CCR7, which is necessary for the homing to secondary lymphoid organs^12^. Interestingly, HIF-1α stabilization in DCs also induces TNF and IL-1β expression.

In this study, we seek to investigate the role of HIF-1α in splenic DCs and to understand how this interferes with the priming and maintenance of protective Th1 responses during chronic VL. Our data demonstrate that HIF-1α hampers IL-12 expression and induces IL-10 in DCs. Moreover, CD11c-specific ablation of HIF-1α results in stronger IFNγ^+^ CD4 T responses and an increased control of parasite growth in the spleen and the bone marrow.

## Results

### Cell-specific ablation of HIF-1α in CD11c^+^ cells increases the recruitment of CD4 T cells to the spleen and enhances Th1 responses

**Figure 1:**
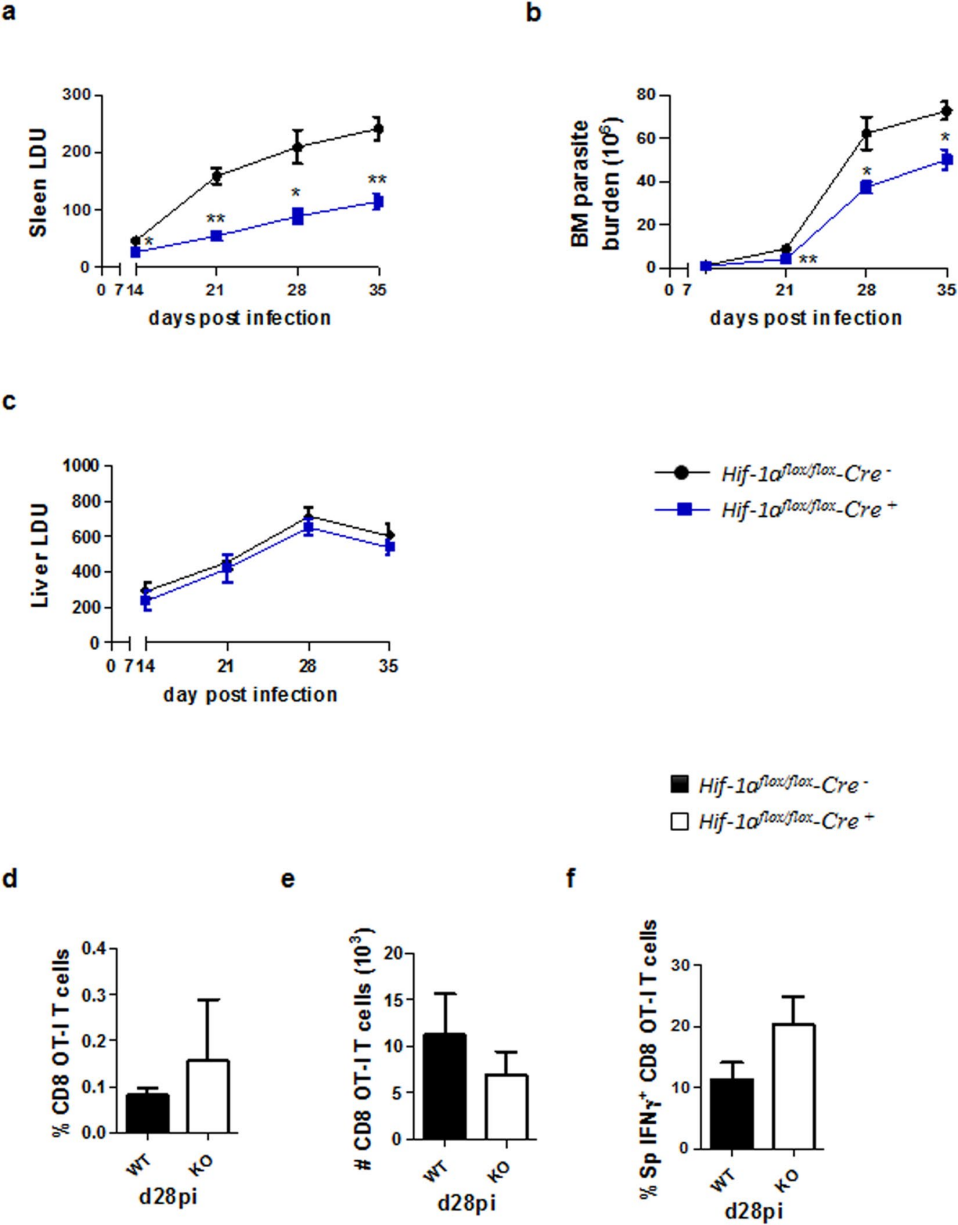
CD11c-specific HIF-1α ablation results in lower parasite burdens in the spleen and bone marrow. *Hif-1α*^*flox/flox*^*Cd11c-Cre*^−^ (WT) and *Hif-1α*^*flox/flox*^*Cd11c-Cre*^+^ (KO) mice were infected with 2 × 10^7^ amastigotes intravenously. (**a**) Graph represents the splenic parasite burden expressed as Leishman Donovan Units (LDU) at various time points after infection. (**b**) Bone marrow parasite burden, determined by limiting dilutions. (**c**) Liver parasite burden (LDU). (**d**–**f**) 2 × 10^4^ OT-I CD8 T cells were adoptively transferred into recipient mice a day prior to infection with ovalbumin-transgenic (PINK) *L. donovani* amastigotes. (**d**) Graph represents the percentage of OT-I CD8 T cells found in the spleen from *Hif-1α*^*flox/flox*^*Cd11c-Cre*^−^ and *Hif-1α*^*flox/flox*^*Cd11c-Cre*^+^ mice over the course of infection. (**e**) The average number of OT-I CD8 T cells. (**f**) The frequency of IFNγ^+^ OT-I CD8 T cells. All data represent mean ± SEM of one of 3 independent experiments, n = 4.

We have previously demonstrated that HIF-1α is up-regulated and stabilized in splenic CD11c^hi^ DC during acute *Leishmania donovani* infection^13^. Moreover, CD11c-specific HIF-1α deficient mice were highly resistant to infection. Control of parasite growth during the first 14 days of infection in the spleen was dependent on antigen-specific CD8 T cell responses^13^. Because, *L. donovani* infection leads to splenomegaly and chronic inflammation, we next wanted to know if HIF-1α was involved at all in the immune response to the parasite during persistent infection. Interestingly, *Hif*^*flox/flox*^ – *Cd11c cre*^+^ mice showed a significantly lower splenic (^13^, and Fig. 1a) and bone marrow (Fig. 1b) parasite burden compared to their *Cre*^−^ littermates, suggesting that HIF-1α in DCs may play an important role during chronic VL as well. No differences between both groups were observed in the liver parasite load (Fig. 1c). In contrast to the acute phase, control of parasite growth was not associated to improved CD8 T cell responses. Indeed, no differences were observed in the recruitment to the spleen (Fig. 1d and e) or in the production of IFNγ (Fig. 1f) by adoptively transferred CD45.1-OT-I CD8 T cells in *Hif*^flox^^*/flox*^ – *Cd11c Cre*^+^ and *Cre*^−^ mice infected with ovalbumin-transgenic *L. donovani*.

Hence, we investigated whether CD4 T cells were involved in increased parasite clearance in CD11c-specific HIF-1α-deficient mice. CD4 T cells are known to play a protective role during VL by producing IFNγ, a cytokine capable of inducing leishmanicidal capacities in macrophages^14^.

**Figure 2:**
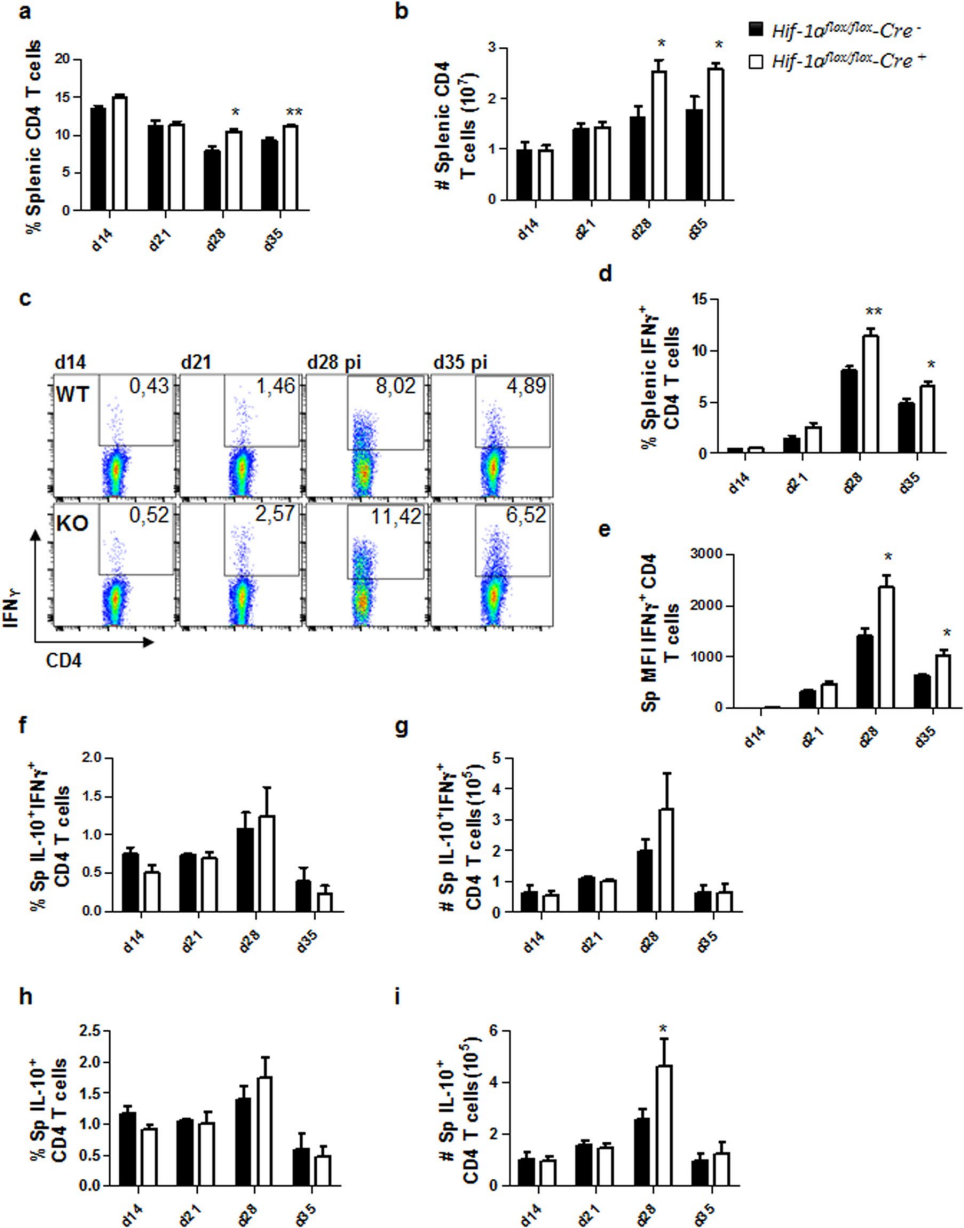
HIF-1α expression in CD11c^+^ cells limits expansion of Th1 cells during chronic visceral leishmaniasis. *Hif-1α*^*flox/flox*^*Cd11c-Cre*^−^ (WT) and *Hif-1α*^*flox/flox*^*Cd11c-Cre*^+^ (KO) mice were infected with 2 × 10^7^ amastigotes intravenously. (**a**) Graph represents the frequency and (**b**) absolute numbers of splenic CD4 T cells. (**C**) Representative FACS plots for IFNγ^+^CD4 T cells of *Hif-1α*^*flox/flox*^*Cd11c-Cre*^−^ (upper panels) and *Hif-1α*^*flox/flox*^*Cd11c-Cre*^+^ mice (lower panels) in the spleen. (**d**) Percentage and (**e**) mean fluorescence intensity (MFI) of IFNγ^+^CD4 T cells in the spleen. Frequency (**f**) and numbers (**g**) of IFNγ^+^IL-10^+^ double producing CD4 T cells; percentage (**h**) and numbers (**i**) of IL-10^+^ single producers CD4 T cells in the spleen over the course of infection. All data represent mean ± SEM of one of 3 independent experiments, n = 4.

We first assessed the frequency and numbers of CD4 T cells present in the spleen between d14 and 35 p.i.. As shown in Fig. 2a and b, CD4 T cells were present at significantly higher frequencies (Fig. 2a) and numbers (Fig. 2b) at d28 and 35 p.i. in the spleen of *Cre*^+^ mice compared to *Cre*^−^ controls. When we examined splenic IFNγ^+^ CD4 T cells, we found that *Hif*^*flox/flox*^ – *Cd11c Cre*^+^ showed a significantly higher frequency of IFN-γ-producing CD4 T cells at d28 and 35 p.i. compared to their *Cre*^−^ littermates (Fig. 2c and d). Moreover, CD4 T cells from HIF-1α conditional knock-outs appeared to produce higher amounts of IFNγ compared to HIF-1α-sufficient mice (Fig. 2c and e). These results suggest that the depletion of HIF-1α in CD11c^+^ cells results in more efficient expansion of functional splenic Th1 cells.

Because disease exacerbation has been associated with IL-10 produced by CD4 T cells^15,16^, we next monitored the frequencies and numbers of IL-10-producing CD4 T cells in the spleen. No differences were observed in frequencies and numbers of splenic IFNγ^+^IL-10^+^ double producing CD4 T cells in *Cre*^+^ and *Cre*^−^ mice over the course of infection (Fig. 2f and g). Similar results were obtained when we assessed IL-10 single producers, with exception of d28p.i., when a slight increase in the numbers, but not the frequency, of these cells was observed in *Cre*^+^ mice (Fig. 2h and i).

### HIF-1α deficiency in CD11c^+^ cells results in stronger Th1 responses in the bone marrow

**Figure 3:**
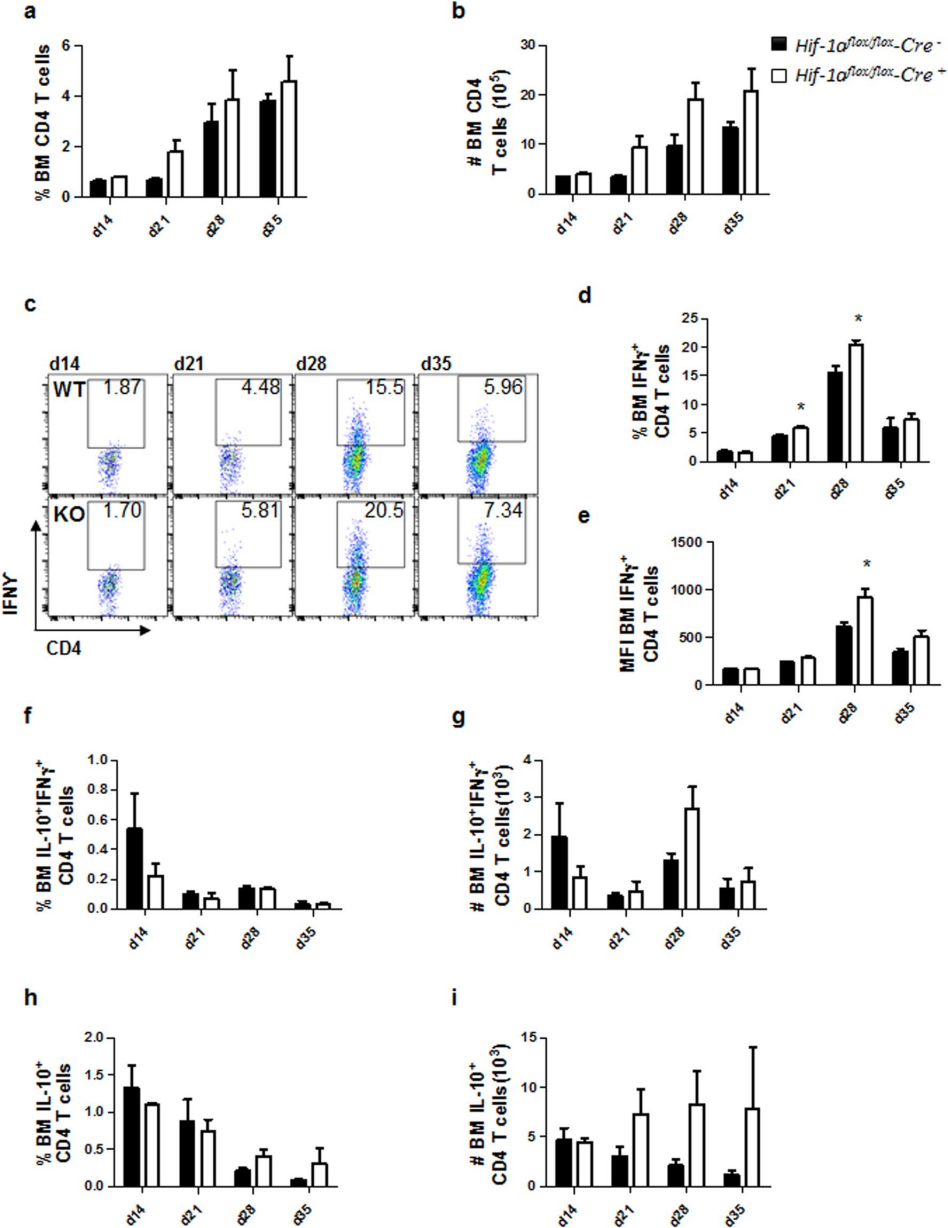
HIF-1α deficiency in CD11c^+^ cells results in stronger Th1 responses in the bone marrow. *Hif-1α*^*flox/flox*^*Cd11c-Cre*^−^ (WT) and *Hif-1α*^*flox/flox*^*Cd11c-Cre*^+^ (KO) mice were infected with 2 × 10^7^ LV9 amastigotes intravenously. (**a**) Graph represents the frequency and (**b**) absolute numbers of bone marrow CD4 T cells. (**c**) Representative FACS plots for IFNγ^+^CD4 T cells of *Hif-1α*^*flox/flox*^*Cd11c-Cre*^−^ (upper panels) and *Hif-1α*^*flox/flox*^*Cd11c-Cre*^+^ mice (lower panels) in the bone marrow. (**d**) Percentage and (**e**) mean fluorescence intensity (MFI) of IFNγ^+^CD4 T cells in the bone marrow. Frequency (**f**) and numbers (**g**) of IFNγ^+^IL-10^+^ double producing CD4 T cells; percentage (**h**) and numbers (**i**) of IL-10^+^ single producers CD4 T cells in the bone marrow over the course of infection. All data represent mean ± SEM of one of 3 independent experiments, n = 4.

Because *L. donovani* establishes chronic infection in the bone marrow (BM) and *Hif*^*flox/flox*^ – *Cd11c Cre*^+^ bared fewer parasites at this site (Fig. 1b) we next monitored the recruitment of CD4 T cells to the BM. Interestingly, the percentage and the number of CD4 T cells constantly increased over the course of infection to reach a peak at d35 p.i. in both groups of mice (Fig. 3a and b). IFNγ^+^ CD4 T cells were also increasingly present in the BM over the course of infection; however, the frequency (Fig. 3c and d) and numbers (Fig. 3c and e) of these cells dropped at d35p.i., as observed in the spleen. Interestingly, higher percentages of IFNγ^+^ CD4 T cells were observed in the BM of mice deficient for HIF-1α in CD11c^+^ cells at d21 and 28p.i. (Fig. 3c and d). In these animals, CD4 T cells also expressed higher amounts of IFNγ at d28 p.i. compared to littermate controls (Fig. 3c and e).

We also monitored IL-10-producing CD4 T cells in the bone marrow of infected mice. Frequencies and numbers of IFNγ^+^IL-10^+^ (Fig. 3f and g) and IL-10^+^ (Fig. 3h and i) were similar in both group of mice over the course of *L. donovani* infection.

Taken together, these results suggest that HIF-1α expression in CD11c^+^ cells may inhibit CD4 T cells recruitment to the bone marrow and/or inhibit the development of Th1 responses during chronic VL.

### Dendritic cell migration to the spleen is not affected by the absence of HIF-1α

**Figure 4:**
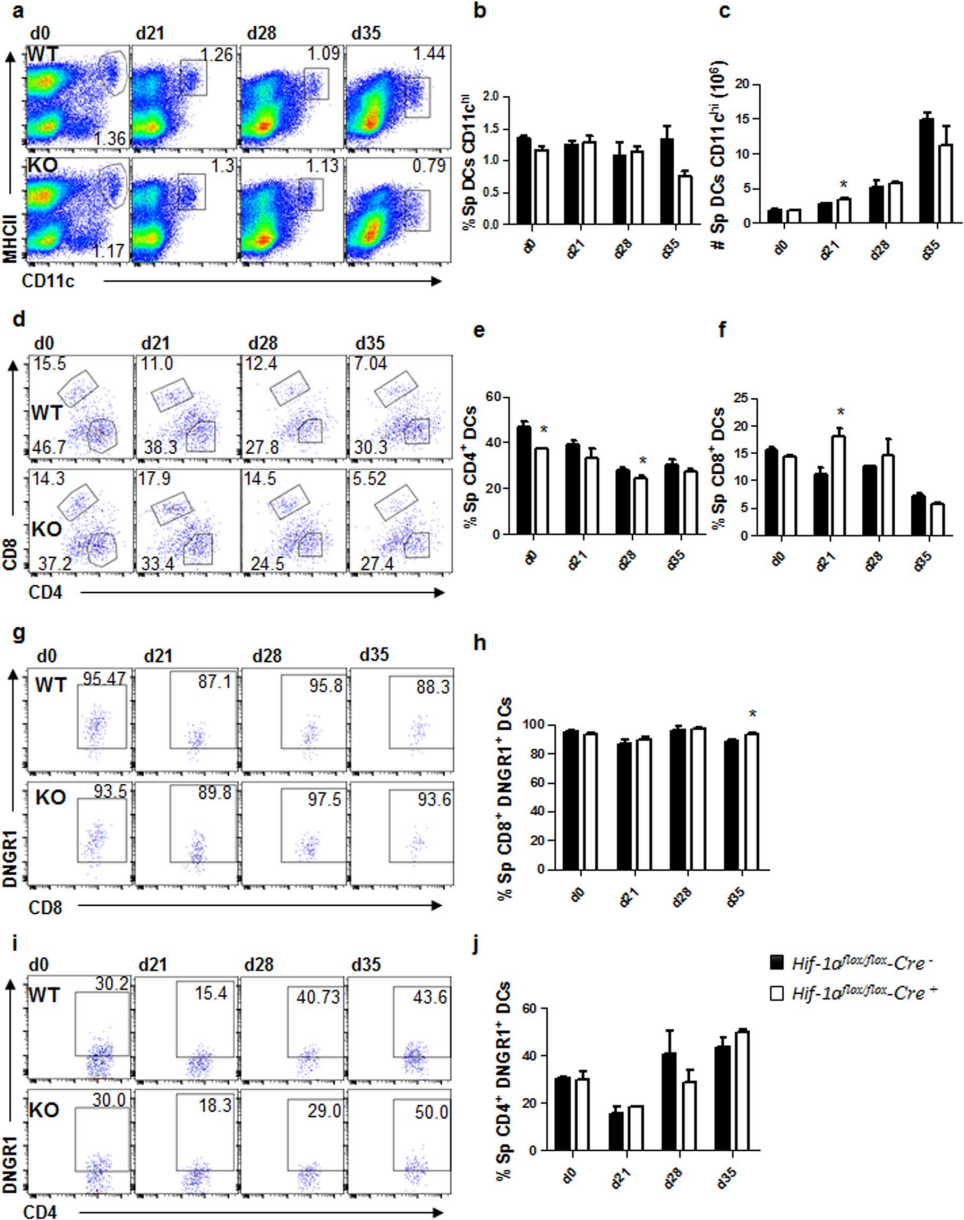
Dendritic cell migration to the spleen is not affected by the absence of HIF-1α. *Hif-1α*^*flox/flox*^*Cd11c-Cre*^−^ (WT) and *Hif-1α*^*flox/flox*^*Cd11c-Cre*^+^ (KO) mice were infected with 2 × 10^7^ LV9 amastigotes intravenously. (**a**) Representative plots showing splenic CD11c^hi^ DCs of *Cre*^−^ (upper panels) and *Cre*^+^ (lower panels) mice over the course of infection. Graphs represent the frequency (**b**) and absolute numbers (**c**) of CD11c^hi^MHCII^hi^ cells in the spleen. (**e,f**) Frequency of splenic CD11c^hi^MHCII^hi^CD4^+^ (**d**) and CD11c^hi^MHCII^hi^CD8^+^ (**e**) cells. (**g,h**) Representative plots (**g**) and percentages (**h**) of DNGR-1^+^ CD8^+^ dendritic cells. (**h,i**) Representative plots (**i**) and percentages (**j**) of DNGR-1^+^ CD4^+^ dendritic cells. All data represent mean ± SEM of one of 3 independent experiments, n = 4.

IL-12-producing DCs are responsible for priming IFNγ-secreting CD4 T cells during experimental VL^17^. Because stronger Th1 responses were observed in the absence of HIF-1α in CD11c^+^ cells and HIF-1α is known to regulate CCR7 expression and myeloid cell migration^9,18,19^, we compared the phenotype and frequency of conventional CD11c^hi^ splenic DCs of *Hif1a*^*flox/flox*^ - *Cd11c-Cre*^+^ and *Cre*^−^ mice during *L. donovani* infection. Conventional splenic DCs were defined as CD11c^hi^MHCII^hi^ cells (Fig. 4a). No major differences were observed in the frequency (Fig. 4b) and numbers (Fig. 4c) of total splenic CD11c^hi^ DCs between both experimental groups. Interestingly, when we analysed the expression of CD4 and CD8 in CD11c^hi^ DCs (Fig. 4d), we noticed that the percentage of CD11b^+^CD4^+^ DCs was slightly reduced in *Cre*^+^ mice (Fig. 4e), while the frequency of CD8^+^ DCs was lower in *Cre*^−^ control mice (Fig. 4f).

We next determined the origin of splenic CD11c^hi^ DC by monitoring DNGR-1 surface expression over the course of infection. As expected, about 85–90% of the CD8^+^ CD11c^hi^ DC population expressed DNGR-1, suggesting that these cells were bone marrow descendants^20^ (Fig. 4g and h). In contrast, the percentage of DNGR-1^+^ CD4^+^ DCs varied during the course of infection. About 30% of CD4 T cells were DNGR-1^+^ in naïve mice of both groups; this percentage decreased during the first 3 weeks of infection to increase again at d28 p.i. (Fig. 4i and j), suggesting that the majority of CD4^+^ DCs derived from monocytes during acute VL. No differences were observed between *Cre*^+^ and *Cre*^−^ mice.

Taken together, our results show that the absence of HIF-1α expression in DCs did not majorly affect their migration from the bone marrow or the differentiation from monocytes. Hence, the stronger Th1 responses observed in *Cre*^+^ compared to *Cre*^−^ mice may be a consequence of improved DC functions.

### HIF-1α expression alters DC functions

**Figure 5:**
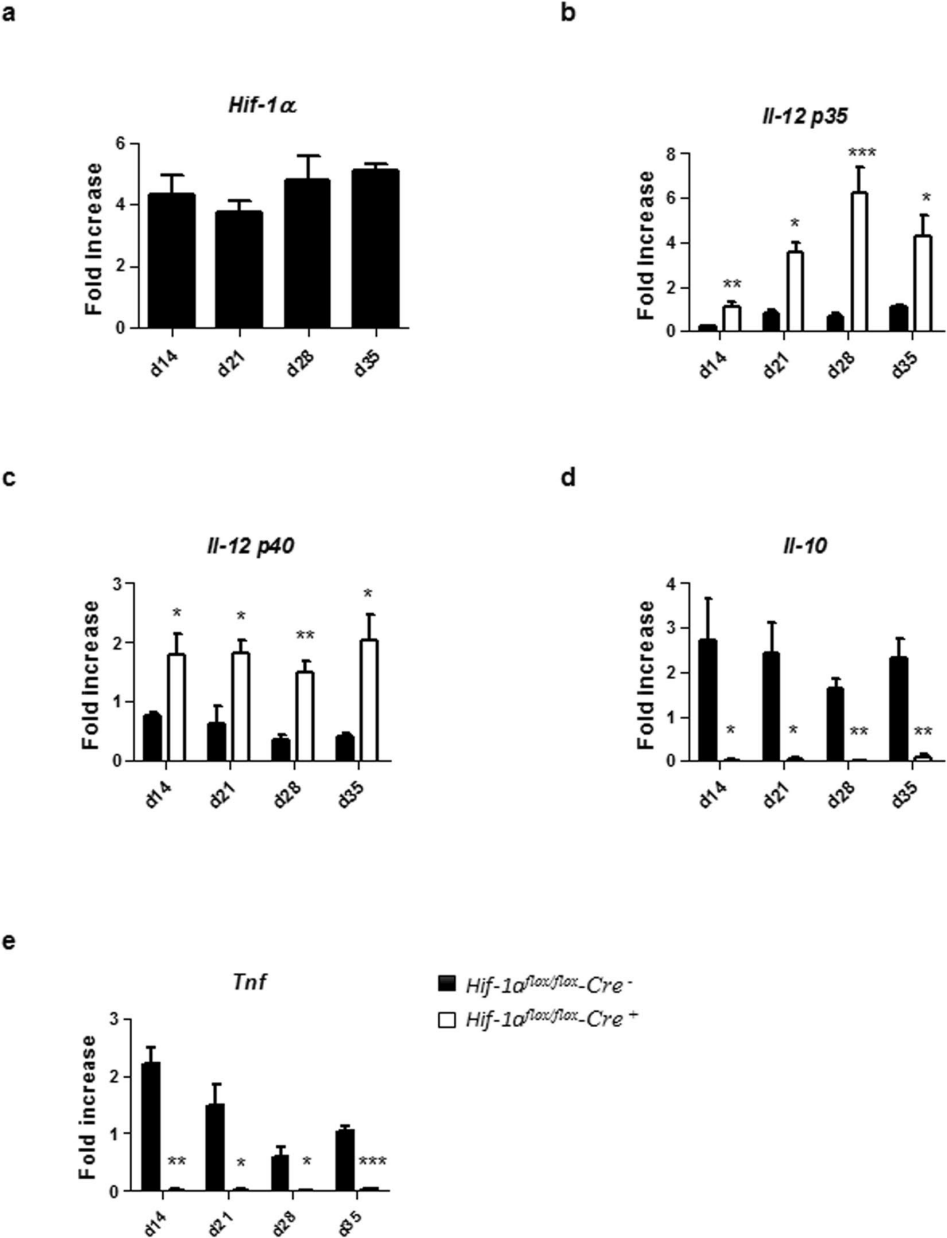
HIF-1α expression alters DC functions. Mice were infected with 2 × 10^7^ amastigotes intravenously. Real-time PCR analysis of HIF-1α (**a**), IL-12p35 (**b**), IL-12p40 (**c**), IL-10 (**d**), and TNF (**e**) expression in splenic DCs from *Hif-1α*^*flox/flox*^*Cd11c-Cre*^−^ (WT) and *Hif-1α*^*flox/flox*^*Cd11c-Cre*^+^ (KO) mice over the course of infection. Gene fold increase was referred to *Hprt* reporter gene. All data represent mean ± SEM combined from 2 independent experiments.

We next assessed if HIF-1α was expressed in splenic DCs from d14 to d35 p.i. and whether HIF-1α expression would alter DC functions. As shown in Fig. 5a, splenic CD11c^+^ cells had already upregulated HIF-1α at d14 p.i.; this upregulation was sustained during chronic VL. Next, we evaluated the expression of IL-12p35 and IL-12p40 by CD11c^+^ cells isolated from the spleen of *L. donovani* infected mice at various time points p.i. As shown in Fig. 5b, IL-12p35 expression was sustained over the course of infection in *Hif1a*^*flox/flox*^ – *Cd11c-Cre*^+^ mice compared to the *Cre*^−^ control group. Similar results were obtained when we measured IL-12p40 (Fig. 5c), suggesting that HIF-1α induction in splenic DCs hampered IL-12 expression.

We also measured the expression of IL-10, a cytokine known to be associated with disease exacerbation^21^. No IL-10 mRNA accumulation was detected in infected HIF-1α deficient CD11c^+^ cells (Fig. 5d); in contrast, HIF-1α-sufficient CD11c^+^ cells continuously expressed this cytokine (Fig. 5d). Interestingly, splenic CD11c^+^ cells lacking HIF-1α also failed to upregulate TNF mRNA levels compared to their HIF-1α-sufficient controls (Fig. 5e). Baseline mRNA levels for HIF-1α and all cytokine in naïve DCs of both groups of mice can be found in Supplemental Fig. 1A–E.

Taking together, our results suggest that the induction of HIF-1α in splenic DCs during chronic VL inhibits IL-12 while inducing IL-10 expression. The impairment in the balance between the two cytokines may lead to inefficient priming of protective Th1 responses.

## Discussion

The transcription factor HIF-1α is known to regulate the function and the migratory capacity of myeloid cells^9,10,22^. However, its effect varies from model to model. The current study demonstrates that HIF-1α stabilization in splenic DCs results in the downregulation of IL-12 with concomitant upregulation of IL-10 expression. CD11c-specific ablation of HIF-1α led to stronger IFNγ^+^ CD4 T responses and increased control of parasite growth in the spleen and the bone marrow.

The microenvironment, especially in chronically inflamed tissues, is involved in shaping the immune response. Although inflammation is crucial for inducing and sustaining effector T cell responses^6^, it can also have adverse effects. Chronically infected organs are highly hypoxic, a condition that promotes the stabilization of HIF-1α. Visceral leismaniasis represents an ideal model to study the effect of chronic inflammation on the immune response to a pathogen. Indeed, *L. donovani* establishes persistent infection in the spleen and bone marrow. This is paralleled by a dramatic enlargement of the spleen, which is more prominent after d21 p.i.. Following infection, high levels of IL-6 and TNF are induced by the parasite^13,23^. Although TNF is essential to clear infection in the liver, it was also reported to be responsible for the disruption of the splenic microarchitecture, which has severe consequences, among other, on T cell migration^24,25^. Tissue disruption together with chronic inflammation typically leads to HIF-1α stabilization^10^. This physiologic response is designed to allow cells to survive under harsh conditions. However, it can also result in immunosuppression^10^.

We have previously reported that HIF-1α expression in DCs resulted in reduced expansion of CD8 T cell responses. CD8 T cells are cross-primed by CD8^+^ DCs, which are mostly bone marrow derived (^20^ and Fig. 4f and g). In this study, we extended our analysis to DCs during the chronic phase of VL, which are mainly monocyte derived (Fig. 4g). HIF-1α depletion in DCs significantly decreased the parasite load in the spleen and bone marrow. This effect could not be attributed to stronger CD8 T cell responses, since CD8 T cells are functionally exhausted during chronic *L. donovani* infection^26^ and no functional differences were observed in both groups of mice. Interestingly, the absence of HIF-1α in DCs directly affected Th1 responses. CD4 T cell responses are first detectable at low levels at d14 p.i.; the peak of response is reached at d28 p.i.^25,27^. In HIF-1α-deficient mice, we not only observed a higher frequency of protective IFNγ^+^ CD4 T cells, but these cells were also producing higher amounts of IFNγ in both target organs, the spleen and the bone marrow. This suggests that Th1 responses were either primed and/or maintained more efficiently in *Cre*^+^ mice.

Interestingly, no major differences were observed in the frequency and numbers of CD11c^hi^ DCs in the spleen of *Cre*^+^ and *Cre*^−^ mice, despite the fact that severe migratory defects have been described for HIF-1α-deficient myeloid cells^9^. Moreover, HIF-1α is known to regulate the expression of CCR7^18,19^, a chemokine that is required for DC migration to the spleen. During experimental VL, DC migration is regulated by the CCR7 ligands CCL19/21, produced by stromal and endothelial cells^28^. However, defective DC migration is observed during chronic infection, due to stromal cell death and IL-10-mediated inhibition of CCR7 expression^12,28^. Thus it is possible that a probable migratory deficit went unnoticed because of the reduced migratory capacity of DCs in infected wild type mice. Nevertheless, HIF-1α-deficient DCs expressed significantly higher levels of IL-12p35 and p40 and lower levels of IL-10 mRNA compared to HIF-1α-sufficient cells. We do not know whether IL-10 expression was directly or indirectly regulated by HIF-1α or is a consequence of a lower parasite burden. Because IL-10 production by T cells was unaltered between infected *Cre*^+^ and *Cre*^−^ mice, perhaps the second hypothesis is the correct one. DC-derived IL-12 is crucial for inducing Th1 responses not only in *Leishmania* infection^29^, but also in other infectious disease models^30–32^. During experimental VL, IL-12 is swiftly induced within 5 h of infection; however, DCs downregulate IL-12 after 24 h and this cytokine expression remains low during the whole course of infection^29^. HIF-1α-deficient DCs not only expressed higher amounts of IL-12, but IL-12 expression was sustained during chronic infection. The exact mechanism by which HIF-1α interferes with DC priming functions is yet unknown. Lawless *et al*. have recently reported that HIF-1α together with mTORC1 and iNOS coordinate DC metabolism and function in pathological microenvironments, limiting DC-stimulated T cell responses. They suggest that glucose is an important signal for the development of T cell responses^33^. Further studies are warranted to link glucose metabolism with IL-12 and IL-10 production by DCs.

In conclusion, our results highlight the detrimental role of HIF-1α in DC functions during chronic VL. The microenvironment characterized by chronic inflammation and hypoxia stabilizes HIF-1α; this is very advantageous for *L. donovani* survival^8,34^ and concomitantly limits the development of protective Th1 responses, resulting in the establishment of chronic infection. Hence, HIF-1α represents a possible therapeutic target for the treatment of VL.

## Methods

### Mice and parasites

C57BL/6-*Tg(OT-I)-RAG1*^*tm1Mom*^ mice were purchased from The Jackson Laboratory. Conditional *Hif-1α* knock-out in CD11c^+^ cells were generated as previously described^13^. All mice were housed at the INRS animal facility under specific pathogen-free conditions and used at 6–10 weeks of age. Experiments involving mice were carried out under protocols approved by the Comité Institutionel de Protection des Animaux of the INRS-Institut Armand-Frappier (1510-02, 1602-02). These protocols and all methods were performed in accordance with regulations and guidelines on good animal practice provided by the Canadian Council on animal care. *Leishmania donovani* (strain LV9) was maintained by serial passage in B6.129S7-*Rag1*^*tm1Mom*^ mice, and amastigotes were isolated from the spleens of infected animals. *Hif-1α Cd11c-Cre*^+^ mice and their littermates *Hif-1a*^*flox/flox*^-*Cre*^−^ were infected by injecting 2 × 10^7^ amastigotes intravenously via the lateral tail vein. Splenic parasite burdens were determined by examining methanol-fixed, Giemsa stained tissue impression smears^13^. Bone marrow parasite burden were calculated by limiting dilutions^13^. Data are presented as number of parasites present in the bone marrow of one femur and one tibia or as Leishman Donovan Units (LDU).

### Adoptive transfer of OT-I cells

Ovalbumin-transgenic parasites were a gift from Drs. P. Kaye and D.F. Smith (University of York, UK). Wild type and ovalbumin transgenic *Leishmania donovani* (strain LV9) were maintained by serial passage in B6.129S7-*Rag1*^*tm1Mom*^/J mice, and amastigotes were isolated from the spleens of infected animals. CD45.1-OT-I/RAG1 mice, transgenic for a T cell receptor specific for chicken ovalbumin 257–264 presented by the MHC class I molecule H-2 K^b^, were used as T cell donors. CD8^+^ T cells were enriched from splenocytes of naïve CD45.1-OT-I/RAG1 animals as previously described^26^. 2 × 10^4^ CD45.1- OT-I CD8 T cells were injected into the lateral tail vein of *Hif1α*^*flox/flox*^ *Cd11c-Cre*^+^ mice and their respective *Cre*^−^ littermate controls. Animals were infected the day after with ovalbumin-transgenic *Leishmania donovani* amastigotes.

The following antibodies were used to further characterize the OT-I response: FITC-conjugated anti-CD45.1 antibody and pacific blue-conjugated anti-CD8 (BD Biosciences).

### Flow cytometry

Splenocytes and bone marrow cells from infected mice were analysed by flow cytometry. Cells were stained with PE-conjugated anti-DNGR-1, APC-conjugated anti-CD11c, FITC-conjugated anti-MHCII and CD4, Pacific blue-conjugated anti-CD8, and Percp-conjugated anti-CD3 (all obtained from eBioscience), as previously described^13^. For intracellular staining, cells were stimulated with BMDC pre-incubated with fixed parasite. 2 hours later, Brefeldin A was added and cells were incubated for further 4 hours. After fixation, cells were permeabilised and stained with APC-conjugated anti-INFγ. Flow cytometric analysis was performed with a *BD* LSRFortessa™ cell analyser (Becton Dickinson). 5.10^5^ cells per sample were acquired and analysed with the FACSDiva or with the flowjo software.

### Real-time PCR analysis

Real-time PCR (Stratagene mx3005p Real time PCR System) was used to analyse transcripts levels of HPRT, IL-10, IL-12p35, IL-12p40, and TNF^13^. CD11c^+^ cells from infected mice were purified and enriched as previously described^13^. Total RNA was isolated using RNeasy (Qiagen) to perform real-time RT-PCR. cDNA was prepared using 500 ng of total RNA using the High capacity cDNA Reverse Transcription kit (Bio Rad). Real time PCR was performed using standard cycle of amplification^13^. Gene fold increase was referred to *Hprt* reporter gene and was calculated based on values obtained in DCs from naïve mice of the respective group.

### Statistical analysis

Statistical analysis was performed using a multi-way ANOVA or Student’s t-test with p < 0.05 considered significant. All experiments were conducted independently at least three times. *denotes *p* < 0.05, **denotes *p* < 0.01 and ***denotes p < 0.001.

### Data availability

All data generated or analysed during this study are included in this published article (and its Supplementary Information files).

## Electronic supplementary material

Supplementary Figure 1
