# Supplementary material for: HIF-1α hampers dendritic cell function and Th1 generation during chronic visceral leishmaniasis
Source: Sci Rep. 2018 Feb 22;8:3500. doi: 10.1038/s41598-018-21891-z (PMC5823892; doi:10.1038/s41598-018-21891-z)
Supplement: Supplementary file 1 — Supplementary Figure 1 [file 41598_2018_21891_MOESM1_ESM.pdf]

"HIF-1α hampers dendritic cell function and Th1 generation during chronic visceral leishmaniasis"

Akil Hammami, Belma Melda Abidin, Krista M. Heinonen, and Simona Stäger

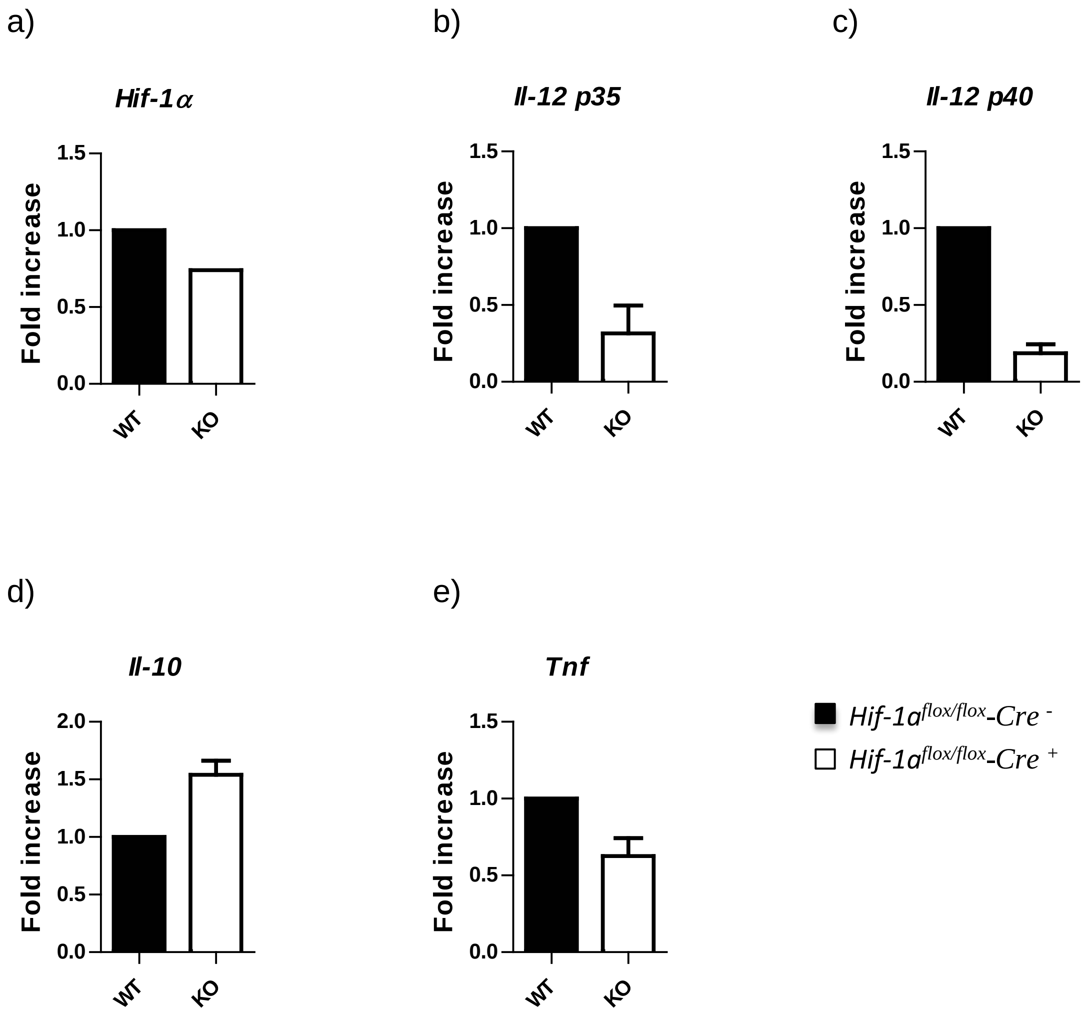

Supplemental Figure 1. Real-time PCR analysis of HIF-1α (A), IL-12p35 (B), IL-12p40 (C), IL-10 (D), and TNF (E) expression in splenic DCs from naive *Hif-1α*$^{flox/flox}$*Cd11c-Cre*$^{-}$ (WT) mice (pool of 3 mice) and *Hif-1α*$^{flox/flox}$*Cd11c-Cre*$^{+}$ (KO) naive mice.
